# Isolation and identification of the *Raoultella ornithinolytica*-ZK4 degrading pyrethroid pesticides within soil sediment from an abandoned pesticide plant

**DOI:** 10.1007/s00203-019-01686-0

**Published:** 2019-06-12

**Authors:** Xiaoqing Zhang, Xiangxiang Hao, Shanshan Huo, Wanzhong Lin, Xinxin Xia, Kuai Liu, Bihua Duan

**Affiliations:** 1 College of Plant Science and Technology, Beijing University of Agriculture/Beijing Key Laboratory of Agricultural Application Technology, No.7 Beinong Road, Changping District, Beijing, 102206 China; 2 The National Agro-Tech Extension and Service Center, No. 20 Maizidian Street, Chaoyang District, Beijing, 100026 China

**Keywords:** Pyrethroid, Strain ZK4, Pesticide-degrading enzyme, Degradation rate, Genome sequencing

## Abstract

We examined how *Raoultella ornithinolytica*-ZK4 degraded pyrethroid pesticides within soil sediment from an abandoned pesticide plant. Lambda-cypermethrin and deltamethrin are two pyrethroid insecticides with high insecticidal activity and a wide range of applications. However, their increased use has raised concerns regarding toxicity and accumulation. We isolated a strain of ZK4 (*Raoultella ornithinolytica*-ZK4) from soil taken from a channel that surrounded a pesticide plant. We used enzyme localization to study degrading bacteria ZK4. The ZK4 strain underwent intracellular enzyme degradation. The degradation rates of lambda-cyhalothrin and deltamethrin were 55% and 53%, respectively. The optimum pH of the two kinds of pyrethroids in ZK4 was 6.5, and their optimum temperature was 37 °C. The intracellular degradation of the crude enzyme produced by the ZK4 strain had a pH of 6.0–8.0 and a temperature of 20–42 °C. The ZK4 strain genome contained 5310 genes with a total length of 4,864,494 bp. Sugar metabolism and exogenous chemical metabolism accounted for the largest proportion of metabolic activities. We used the clusters of orthologous groups (COG) alignment and found numbers for 4686 protein sequences, accounting for 88.25% of the total predicted protein. ZK4 degraded lambda-cyhalothrin and deltamethrin, and may serve as a reference for the preparation of future degrading microbial agents to assist with environmental restoration efforts.

## Introduction

*Pyrethroid* is the second largest class of insecticides, other than organophosphorus pesticides, and accounts for about 20% of the global pesticide market. In China, it is applied to one-third of the total chemical pesticide application area (Wu et al. 2013). Deltamethrin and lambda-cyhalothrin are pyrethroid insecticides with high insecticidal activity. They are used to control various pests and are highly efficient, low cost, and light stable. Deltamethrin accounts for 80.6% of all pesticides used to treat vegetables in Beijing (Huang et al. 2008). However, pyrethroid pesticides have accumulation toxicity, reproductive toxicity, and neurotoxicity. They are highly toxic to individual non-target organisms such as bees and silkworms, and certain aquatic organisms. Consequently, pyrethroid pesticide residue is believed to harm the environment (Sun et al. 2002; Wang et al. 2000; Yin et al. 2011). Strategies for effective use of pyrethroids to reduce environmental damage are, therefore, of considerable interest.

Microbial remediation technology is a popular means of treating pesticide residues. The technology features simple application, a lack of secondary pollution, low energy consumption, and good degradation effects. Degradable pesticide microorganisms are obtained by screening and isolating high-efficiency degradation performance strains from soil contaminated with pesticides, and cultivating effective strains through breeding and engineering (Teng et al. 2007).

There is considerable research on the performance of pyrethroid pesticide-degrading bacteria. These bacteria are obtained from soil contaminated with pyrethroid pesticides, lakes, rivers, and pesticide wastewater. Many microorganisms of different genera have been isolated and tested for long-term degradation performance. For example, (Maloney et al. 1988) found that over 2 weeks, permethrin was degraded 80% by *Bacillus cereus*, 50% by *Pseudomonas fluorescence*, and 75% by *Achromobacter*. After 4 weeks, the degradation rates reached 90% for *Bacillus cereus*, 55% for *Pseudomonas fluorescence*, and 90% for *Achromobacter*.

Magdoub et al. (1989) and Misra et al. (1996) studied fenvalerate in milk and found that it was adsorbed and degraded by lactic acid bacteria, which were then inoculated to the contaminated milk. This process effectively reduced fenvalerate content. *Acidomonas* sp. can degrade pyrethroid by 70% after 72 h of culture in liquid salt medium, with a pyrethroid concentration of 16 mmol/L. Tallur et al. (2008) found a micrococcal strain (*Micrococcus* sp.) that could grow with 1.0 g/L cypermethrin as the sole carbon source. The energy source was isolated from the soil contaminated with pyrethroid insecticides by enrichment culture. Cypermethrin can be completely mineralized by this strain and has good degradation efficiency.

Yu et al. (1996) isolated a strain of *Pseudomonas* sp. YF05 from silkworm sludge. This strain could degrade a variety of pyrethroid pesticides. For example, 50 mg/L of fenvalerate exhibited a degradation rate of 78.6% at 10 h. Further, 100 mg/L methyl parathion reached a degradation rate of 92.2% by 14 h.

*Pseudomonas aeruginosa* GF31 was isolated from cypermethrin-contaminated soil, which can be fixedly grown in the voids of a zeolite carrier. The degradation rate of cypermethrin reached 61.4% (Li et al. 2013).

Xu et al. (2004) isolated a strain of actinomycetes (*Rhodococcus* sp. CDT3) from the sludge of the pesticide plant using an indoor culture. The strain was identified as *Rhodococcus* by 16SrDNA sequencing and 100 mg in 72 h. The degradation rate of cypermethrin was as high as 84.24%.

Liu (2007) isolated a strain of *Pseudomonas aeruginosa* HF12-8 from cypermethrin-contaminated soil. The degradation rates of cypermethrin and bifenthrin, each at a concentration of 20 mg/L, were 93.03% and 58%, respectively.

Wang et al. (2016) isolated a strain of cyhalothrin-degrading fungus from the sewage sludge of the pesticide plant. The fungus was identified by 18SrDNA (*Penicillium* sp.). The degradation rate of lambda-cyhalothrin reached 83.90% over 7 days, with simultaneous degradation of deltamethrin and beta-cypermethrin.

Shi et al. (2016) isolated a strain of *Bacillus licheniformis* BFE-023 which could degrade fenvalerate by enrichment culture in soil sprayed with pyrethroid pesticides. After optimization, the degradation rate of fenvalerate peaked at 88.71% within 60 h. The intermediate product was 3-phenoxybenzoic acid (3-PBA), and pesticide degradation also decreased 3-PBA. These results indicated that the bacteria may also have the ability to degrade intermediates.

Cellular enzymes are the main microbial degraders of pesticides. During biodegradation, the microenvironment often harms microorganisms. Degrading enzymes are directly applied to help eliminate this effect. Compared with the degradation of pesticides by microbial strains, pesticide-degrading enzymes are highly efficient, safe, and feature good environmental tolerance and broad degradation spectrums (Furmanczyk et al. 2017; García et al. 2017; Gouda et al. 2018; Liu et al. 2016; Marican and Duránlara 2018; Chen 2003; Tang. 1988; Xu et al. 2012). Compared with microbial cells, pesticide-degrading enzymes have superior application prospects.

Gas chromatography (GC) has a wide range of applications, high separation efficiency, fast analysis speed, and high sensitivity. These features make it the preferred method for analyzing most thermostable pesticides. It is also the primary method for the analysis and detection of pyrethroid pesticide residues (Zhu 2011). She et al. (2010) successfully detected residual lambda-cyhalothrin in soil using GC-EDC. The minimum-detected concentration was 0.01 mg/kg.

We attempted to isolate and screen bacterial strains that efficiently degraded deltamethrin and lambda-cyhalothrin from soil sediment samples obtained from both sides of the sewage outlet of an abandoned pesticide plant. We identified the properties of these strains and studied the degradation characteristics of a selected strain: ZK4. We further examined the degradation effect of the crude enzyme extract on deltamethrin and lambda-cyhalothrin using GC. Here, we included conditions that affected the degradation of crude enzymes. A bioinformatics analysis was carried out to provide a theoretical basis for constructing efficiently engineered strains, preparing degrading bacteria, and environmental restoration.

## Materials and methods

### Test materials

#### Soil sample collection

Soil samples were collected from the discharge port of an abandoned pesticide factory in Kaifeng City, Henan Province. We collected soil deposits at different distances from the sides of the plant’s sewage outlet and mixed the bags according to the four-point method. The bags were then sealed and returned to the laboratory for storage at 4 °C.

#### Preparation of medium

Enrichment medium, LB medium, LA medium, basal medium, expansion medium, and related physiological and biochemical characteristics determination medium were prepared as per the method described by Zhu (2011). Benzene (chromatographically pure), acetone (analytical grade), standard lambda-cyhalothrin (25 g/L), standard deltamethrin (25 g/L), and other drugs were purchased from Beijing Aoboxing Biotechnology Co., Ltd.

### Test methods

#### Separation and screening of degrading bacteria

Primary screening: simultaneous use of liquid gradient culture and solid plate screening.

The mixed mother liquor preparation, liquid gradient domestication screening method, and solid plate screening method were carried out as per “Modern Microbiology Experimental Technology,” edited by Zhu (2011). We used 50 mg/L of deltamethrin and lambda-cyhalothrin for the mixed mother liquor. For the liquid gradient domestication screening, the amount of deltamethrin and lambda-cyhalothrin were: l00 mg/L (culture 7 days)—150 mg/L (culture 7 days)—200 mg/L and 250 mg/L (cultured 7 days). For the solid plate screening method, we used 10% deltamethrin and lambda-cyhalothrin. Isolated strains were purified three times and double-stored at 4 °C and glycerol − 20 °C for use.

#### Preparation of the bacterial suspension

The bacterial suspension was prepared as per “Modern Microbiology Experimental Technology” (Zhu 2011). The adjusted cell concentration was 1.0 OD_600_.

#### Determination of degradation properties

We determined the degradation performance of each strain as per the sputum and other methods (Cheng et al. 2015) using a Shimadzu GC-2014 gas chromatograph. Accurately absorb 2 mL of the culture solution after 7 days of culture in a 20 mL centrifuge tube, add 1 mL of saturated NaCl solution, and extract three times with 4 mL, 4 mL, 2 mL of petroleum ether, each time vortex for 1 min, wait 15 min. After washing with anhydrous sodium sulfate, the mixture was brought to a total volume of 10 mL with petroleum ether and subjected to GC.

Preparation of standard curve for deltamethrin and lambda-cyhalothrin pesticides: Design mixed standards at concentrations of 10 mg/L, 20 mg/L, 30 mg/L, 40 mg/L, 50 mg/L, Gas chromatograph injection detection, the obtained regression equation through the addition of two kinds of pyrethroids in the water sample recovery test, calculated addition recovery and coefficient of variation.

#### Morphological identification of strains

The morphology of the strain was identified using a McAudi BA310 digital biological microscope. We noted plate colony characteristics and Gram stain and spore staining results.

#### Physiological and biochemical characteristics of strains

Please refer to “Modern Microbiology Experimental Technology,” edited by Zhu (2011).

#### Degradation bacteria growth curve

Please refer to “Modern Microbiology Experimental Technology,” edited by Zhu (2011). The OD_600_ value was measured at a wavelength of 600 nm using an ultraviolet spectrophotometer. The growth curve was plotted with the culture time as the abscissa and the OD_600_ value as the ordinate.

#### Molecular biological identification of the ZK4 strain

16SrDNA was identified for the bacterial strain ZK4: DNA was extracted using the GV-Bacterial Genomic DNA Extraction Kit and amplified using the bacterial universal primer 27F (5′-AGAGTTTGATCCTGGCTCAG-3′), 1492R (5′-TACGGCTACCTTGTTACGACTT-3′). PCR reaction system: 2 × Taq PCR Green Mix 12.5 μL; DNA template 1–5 μL; primer F (10 μm) 0.5 μL; primer R (10 μm) 0.5 μL; nuclease-free water to 25 μL; total volume 25 μL. The PCR product was sent to Beijing Liuhe Huada Gene Sequencing Company and sequenced using an Illumina Hiseq 4000 sequencer. The returned data were analyzed, and the phylogenetic tree constructed as per “Modern Microbiology Experimental Techniques” (Zhu 2011).

#### Activation of the ZK4 strain

Please refer to “Modern Microbiology Experimental Technology,” edited by Zhu (2011).

#### Preparation of the ZK4 strain crude enzyme solution

The activated ZK4 strain was inoculated into an expansion medium that contained 100 mg/L of lambda-cyhalothrin and deltamethrin pesticide, cultured at 37 °C, placed in a 160 r/min shaker for 3 days, then centrifuged at 10,000 r/min. After centrifugation for 10 min, the supernatant and the slime were collected separately. The collected sludge was washed with 0.02 mol/L phosphate buffer (pH 7.0) three times and then suspended in a phosphate buffer solution (3 mL phosphate buffer per 1 g bacteria sludge). The bacterial suspension was then placed on ice and homogenized using a YIY-UL500 W-L ultrasonic homogenizer. The homogenized bacteria were crushed and processed over seven cycles. Each cycle consisted of 10 s processing followed by a 7 s rest. The fully lysed bacterial solution was centrifuged at 4 °C for 10 min at 10,000 rpm, and the supernatant was used as a cell-free intracellular crude enzyme solution.

The activated ZK4 strain was inoculated into an expansion medium containing 100 mg/L of lambda-cyhalothrin and deltamethrin pesticide, and cultured at 37 °C, shaken at 160 r/min for 3 days, then centrifuged at 10,000 r/min. After centrifugation for 10 min, we separately collected the supernatant and cells. The supernatant was placed on ice, and ammonium sulfate was added until the saturation was 100%. After salting out at 4 °C overnight, the precipitate was collected by centrifugation at 10,000 r/min for 10 min. After dissolving with a small amount of 0.02 mol/L phosphate buffer (pH 7.0), dialyzing with the same buffer (MWCO 12,000–14,000), and using barium chloride to confirm there was no SO_4_^2−^, an extracellular crude enzyme solution was obtained.

#### Determination of ZK4 enzyme activity

We added 2 mL of crude pre-heated ZK4 degradation enzyme solution to 18 mL of buffer (pH 7.0) containing both 100 mg/L lambda-cyhalothrin and 100 mg/L deltamethrin. The mixture was placed in a constant temperature water bath at a specific temperature for 3 h. After this, we added 0.5 mL of a 1.0 mol/L HCl solution to terminate the reaction. Three parallels were set for each treatment, and a buffer of two pyrethroid pesticides without a crude enzyme solution was used as a control. After the end, accurately absorb 2 mL of the culture solution, and then extract three times with 4 mL, 4 mL, 2 mL of n-hexane, each time vortex for 1 min, wait 15 min. After washing with anhydrous sodium sulfate, we adjusted the volume to 10 mL with petroleum ether. Residual pesticide was detected by gas chromatography, after which we calculated the pesticide degradation rate.

#### Establishment of a pyrethroid pesticide detection method

Shimadzu GC-2014 Gas Chromatograph Gas Chromatography Conditions: RTX-5 capillary column (30 m, 0.25 mm, 0.25 μm); inlet temperature: 250 °C, column flow rate: 1.0 mL/min; column temperature: maintain at 250 °C; split injection: 1:20, injection volume: 1.0 μL; ECD detector: 280 °C. Qualitative retention time, peak area quantified.

#### Calculation of degradation rate of pyrethroid pesticides

$${\text{Pyrethroid}}\;{\text{degradation}}\;{\text{rate}} = \frac{{{\text{Blank}}\;{\text{sample}}\;{\text{content}} - {\text{control}}\;{\text{sample}}\;{\text{content}}}}{{{\text{Blank}}\;{\text{sample}}\;{\text{content}}}} \times 100\%$$

#### Effect of pH on the activity of the ZK4 strain for degradation of crude enzyme

We stored 200 μL of the crude enzyme solution and phosphate buffer at pH levels of 4.0, 5.0, 6.0, 6.5, 7.0, 8.0, and 9.0 (containing beta-cyhalothrin, deltamethrin pesticide) at 30 °C for 3 h. The reaction was terminated using 0.2 mL of a 1.0 mol/L HCl solution.

#### Effects of different temperatures on the degradation of crude enzymes

We incubated 200 μL of phosphate buffer containing crude enzyme solution at a pH of 7.0 at 4 °C, 20 °C, 30 °C, 37 °C, 42 °C, 60 °C, 80 °C for 3 h. We then added lambda-cyhalothrin and deltamethrin pesticide (pesticide concentration is 100 mg/L). The reaction was carried out at 37 °C for 3 h, and 0.2 mL of 1.0 mol/L HCl solution was added to terminate the reaction.

## Results and analysis

### Isolation and identification of pyrethroid pesticide-degrading bacterial strains in soil

#### Isolation of strains and determination of degradation rate

Taking deltamethrin and lambda-cyhalothrin as research objects, three strains of bacteria capable of effectively degrading two pyrethroid pesticides were obtained by enriching, separating, and purifying the strains in soil samples. These strains were *Raoultella ornithinolytica* ZK4 (accession number: KJ806387.1), *Pseudomonas* sp. ZF1, and *Enterobacter* sp. ZW3. ZK4, as a bacterium with the ability to degrade deltamethrin and lambda-cyhalothrin, has not been the focus of previous reports.

Using *Pseudomonas aeruginosa* (KB) as a control, we determined the degrading rates of two pyrethroid pesticides under identical conditions for each of the selected three bacterial strains. The results are shown in Table 1. The degradation rate of the newly discovered strain ZK4 to lambda-cyhalothrin was 54%, which is slightly higher than the degradation rate of *Pseudomonas* sp. ZF1 (52%), and slightly lower than that of *Enterobacter* (*Enterobacter* sp.) The ZW3 degradation rate of 57%; the ZK4 strain degradation rate of deltamethrin 52%, slightly lower than the degradation rate of *Pseudomonas* sp. ZF1 54%, higher than *Enterobacter*. The degradation rate of *Enterobacter* sp. ZW3 was 44%. Therefore, an in-depth study of the ZK4 strain was necessary.

**Table 1:** Degradation rates of three bacterial strains

	KB	ZF1	ZW3	ZK4
High efficiency lambda-cyhalothrin content (mg/L)	70.45	37.18	33.30	35.86
Degradation rate (%)	0	52	57	54
Content of deltamethrin (mg/L)	80.95	37.24	45.33	38.86
Degradation rate (%)	0	54	44	52

#### Electrophoresis detection of PCR products of the ZK4 strain and construction of a phylogenetic tree

**Fig. 1:**
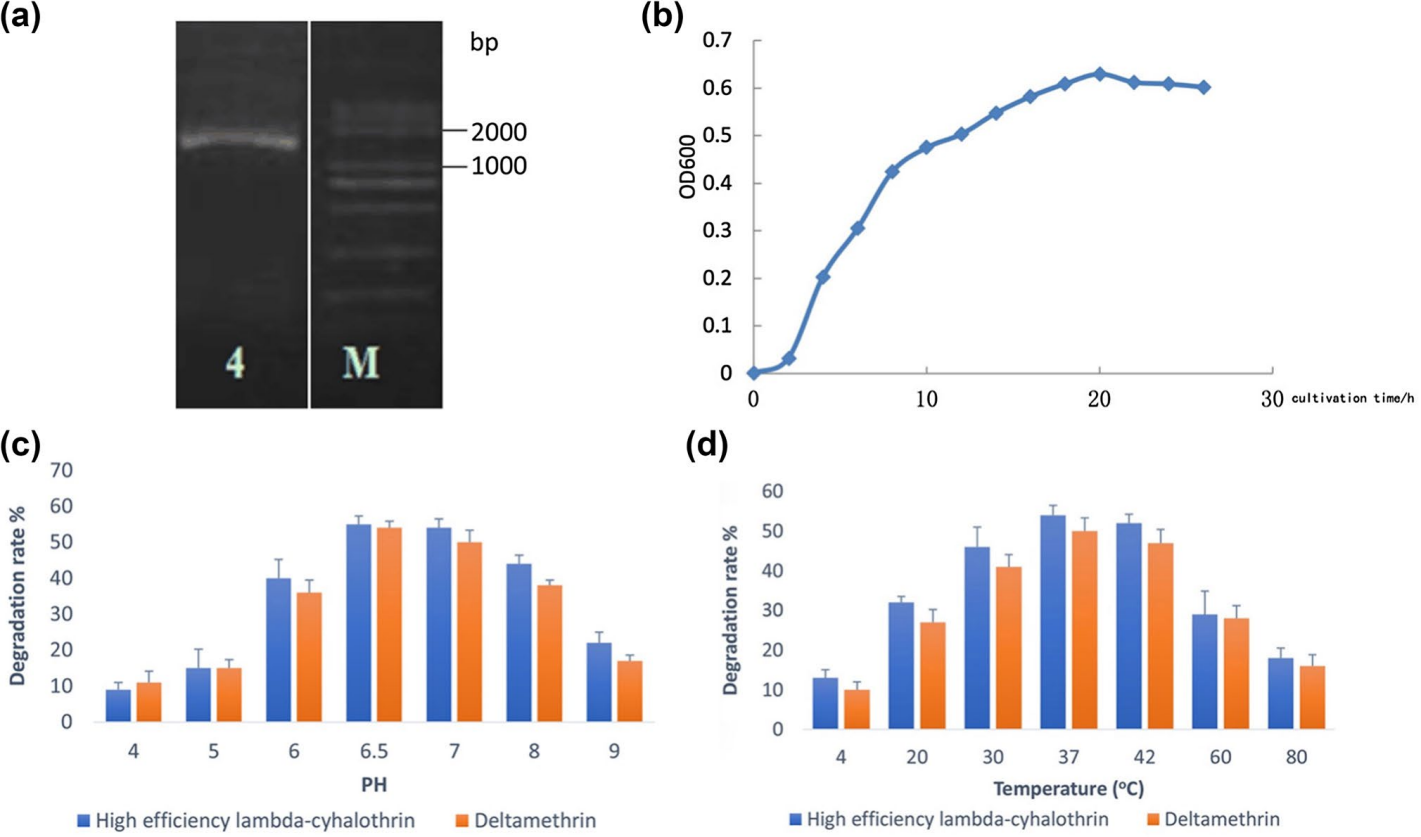
The result of the ZK4 PCR, the growth curve of the ZK4 strain and effect of pH and temperature on the degradation performance and stability of endoenzymes. **a** The results of the ZK4 PCR; **b** The growth curve of the ZK4 strain; **c** Effect of pH on the degradation performance of endoenzymes; **d** Effect of temperature on the degradation performance of endoenzymes

Using the total primer of the ZK4 strain as a template, the universal primer of the 16SrDNA gene was subjected to PCR amplification to obtain a clear target band of about 1500 bp (Fig. 1a). The fragment was recovered, and its size was found to be 1414 bp based on the sequencing result.

**Fig. 2:**
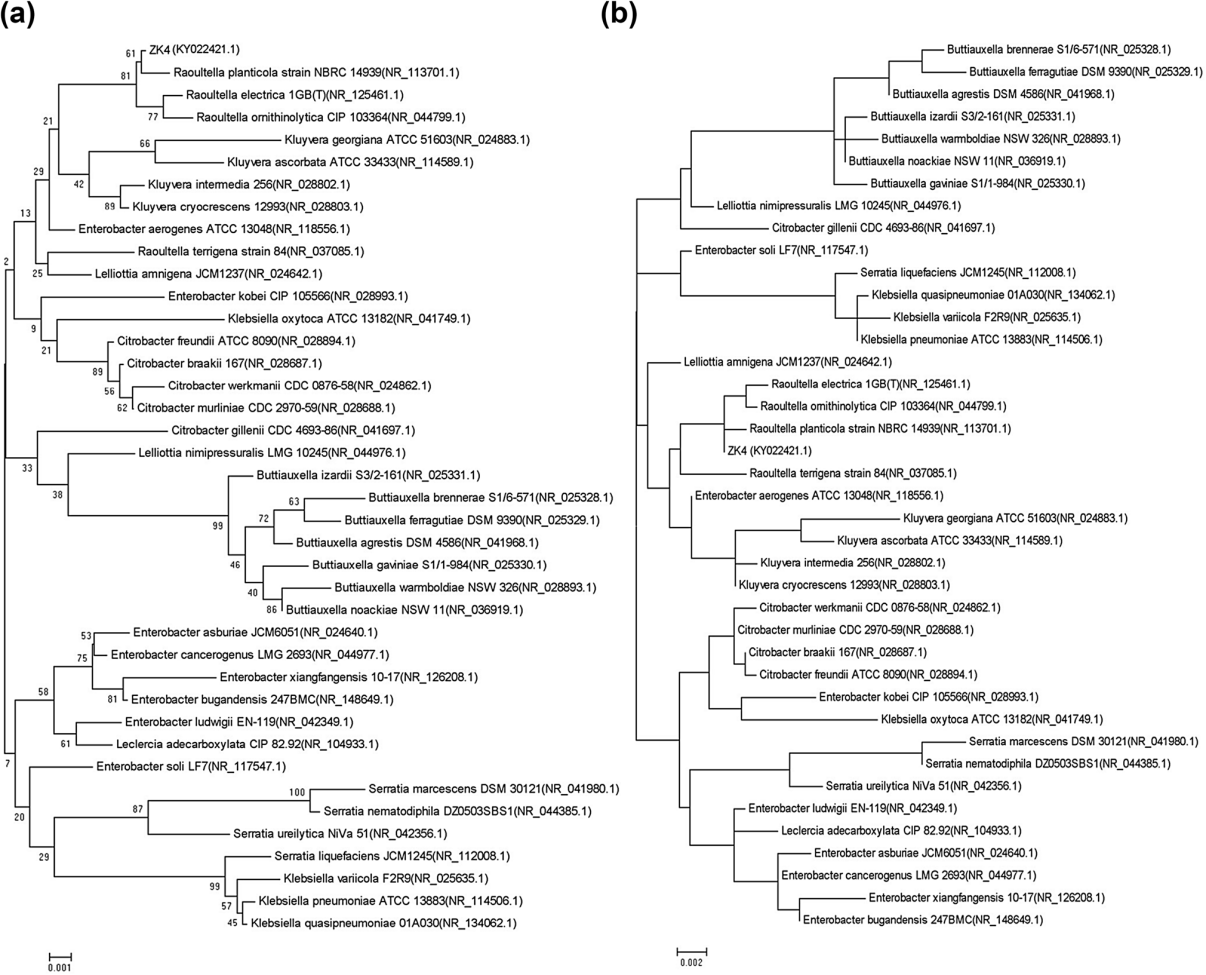
The phylogenetic tree of the ZK4 strain for NJ and ML. **a** The phylogenetic tree of the ZK4 strain for NJ; **b** The phylogenetic tree of the ZK4 strain for ML

The sequence homology was compared with the sequence in GenBank. The similarity between the 16SrDNA sequence of the ZK4 strain (accession number: KY022421.1) and *Raoultella ornithinolytica* KJ806387.1 was up to 99% homologous. The similarities among the 16SrDNA sequences of *Raoultella planticola* ATCC 335, *Raoultella terrigena* ATCC 3325, and *Klebsiella pneumoniae* KM096433.1 were also high. To determine the phylogenetic position of the ZK4 strain, some *Raoultella* were selected to construct a phylogenetic tree, with *Enterobacter* selected as the outer group. The names and the serial numbers of each strain are shown in Fig. 2a, b.

#### Morphological identification of the ZK4 strain

**Fig. 3:**
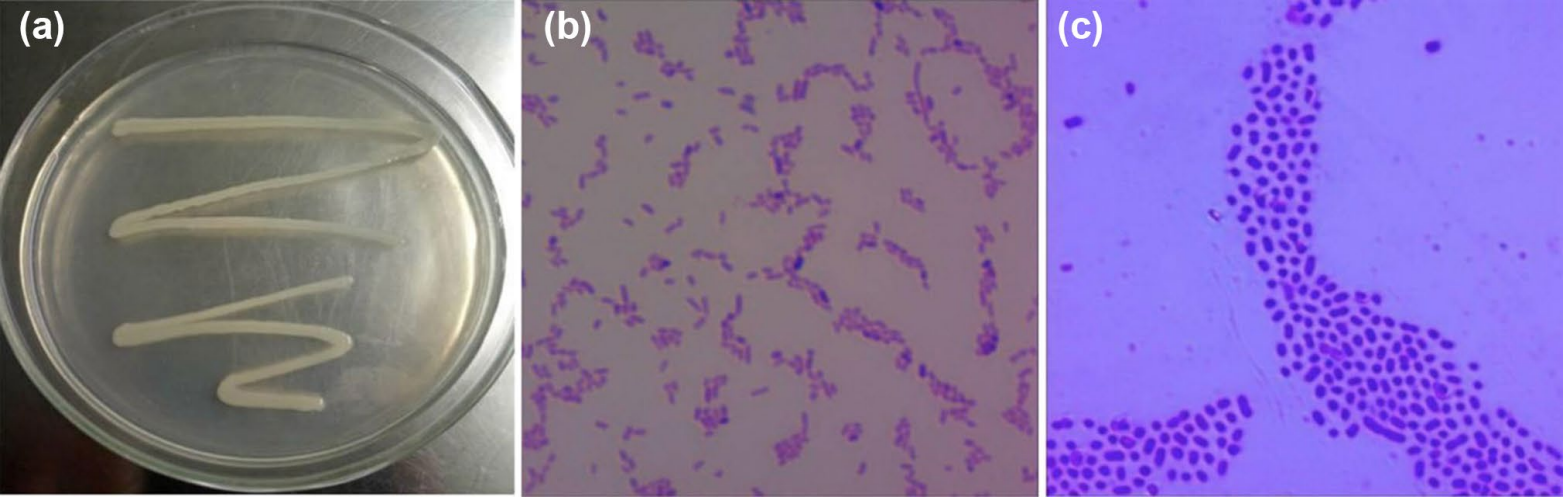
Characterization of the ZK4 strain. **a** The morphological characteristics of ZK4 colonies after 24 h of culture; **b** ZK4 Gram stain (100×); **c** ZK4 Capsule stain (100×)

Figure 3a–c shows the morphology of the colony after application of the ZK4 strain for 24 h, Gram staining, and capsular staining results. The ZK4 strain was cultured for 24 h, revealing a colony that was smooth and milky white, with a moist, shiny, and irritating surface. After Gram staining, the bacteria were pink, and the Gram stain was negative. The bacteria were blue, and the capsule was pink under a blue-violet background.

#### Physiological and biochemical characteristics of the ZK4 strain

The physiological and biochemical identification results for the ZK4 strain are shown in Table 2. The strain ZK4 starch hydrolysis test, gelatin liquefaction test, casein hydrolysis test, catalase test, phenylalanine deaminase, and hydrogen sulfide test were all negative. The methyl red experiment, glucose oxidation fermentation experiment, nitrate reduction test, the sputum test, and the urease test showed positive reactions. The ZK4 strain performed like the control strain *Pseudomonas aeruginosa* during the gelatin liquefaction test, the glucose oxidative fermentation test, and the nitrate reduction test.

**Table 2:** The physiological and biochemical properties of ZK4

Physiological and biochemical characteristics	*Pseudomonas aeruginosa* (ck)	*Raoultella ornithinolytica* (ZK4)
Starch hydrolysis test	+	–
Gelatin liquefaction	–	–
Methyl red test	–	+
Oxidation and fermentation of glucose	+	+
Casein hydrolysis	+	–
Catalase test	+	–
Phenylalanine deaminase test	+	–
Nitrate reduction test	+	+
Indole test	–	+
Hydrogen sulfide test	+	–
Urease test	–	+

#### Growth curve of the ZK4 strain

The sample obtained under aseptic processing conditions was placed in an ultraviolet spectrophotometer to determine the OD_600_ value at a wavelength of 600 nm. The growth curve was established by taking the OD_600_ value as the ordinate and the culture time as the abscissa (Fig. 1b). The growth retardation period of the ZK4 strain was 0–2 h and caused by the bacteria needing to adapt to their new environment. During this time, the ZK4 strain exhibited enlarged cells and active metabolic capacity. It accumulated sufficient enzymes, coenzymes, and intermediate metabolites for the subsequent bacterial division and reproduction; however, cell division during this time was slow, and there were fewer bacterial cells in the liquid medium. During the logarithmic phase of the growth of the strain from 4–16 h, the bacteria proliferated due to the accumulation of growth-promoting substances in the lag phase. The number of viable cells increased geometrically, and the number of bacteria increased linearly, eventually peaking. The morphology, staining, and physiological activity of the bacteria during this period appeared typical. After 18 h, the bacterial growth gradually slowed down and stabilized, then began to decay. Due to the consumption of nutrients in the medium, the bacterial growth rate gradually slowed, and the number of dead bacteria increased.

### Location of crude enzymes for pyrethroid pesticide degradation

After the strain ZK4 was cultured for 3 days in an expansion medium containing lambda-cyhalothrin and deltamethrin pesticide, the intracellular crude enzyme solution was obtained by centrifugation and cell disruption. The extracellular crude enzyme solution was obtained by ammonium sulfate precipitation. The degradation properties of the two kinds of pyrethroid pesticides in the cell crude enzyme solution and extracellular crude enzyme solution were determined and the results are shown in Table 3.

**Table 3:** Degradation of pesticides by endoenzymes

	Lacy-cyhalothrin degradation rate (%)	Deltamethrin degradation rate (%)
Intracellular crude enzyme solution	55	53
Extracellular crude enzyme solution	5	–

The degradation rates of the intracytosolic enzyme to lambda-cyhalothrin (55%) and deltamethrin (53%) both exceeded 50%, while the extracellular enzyme was on high-efficiency cyhalothrin. The ester degradation rate was only 5%. The degradation of the two pyrethroid pesticides by the ZK4 strain was attributable to intracellular enzyme degradation.

### Determination of intracellular enzyme properties

We examined the effects of pH and temperature on endoenzyme activity in ZK4 (Fig. 1c, d). Degradation was observed within a pH range of 4.0–9.0. Specifically, the highest degradation rate of 53–57% was observed within the pH range of 6.5–7.0, when the degradation rates of high-efficiency lambda-cyhalothrin and deltamethrin were 57% and 55%, respectively. The two kinds of pyrethroid pesticides produced by the ZK4 strain had degradation effects at temperatures ranging from 4 to 80 °C. Given the good degradation rates that were demonstrated between 30 and 42 °C, we determined that this was the optimum temperature range. As seen in Fig. 1d, the optimum temperature was 37 °C, at which the degradation rates were 54% for lambda-cyhalothrin and 50% for deltamethrin.

### Analysis of the ZK4 strain genome sequencing data

**Fig. 4:**
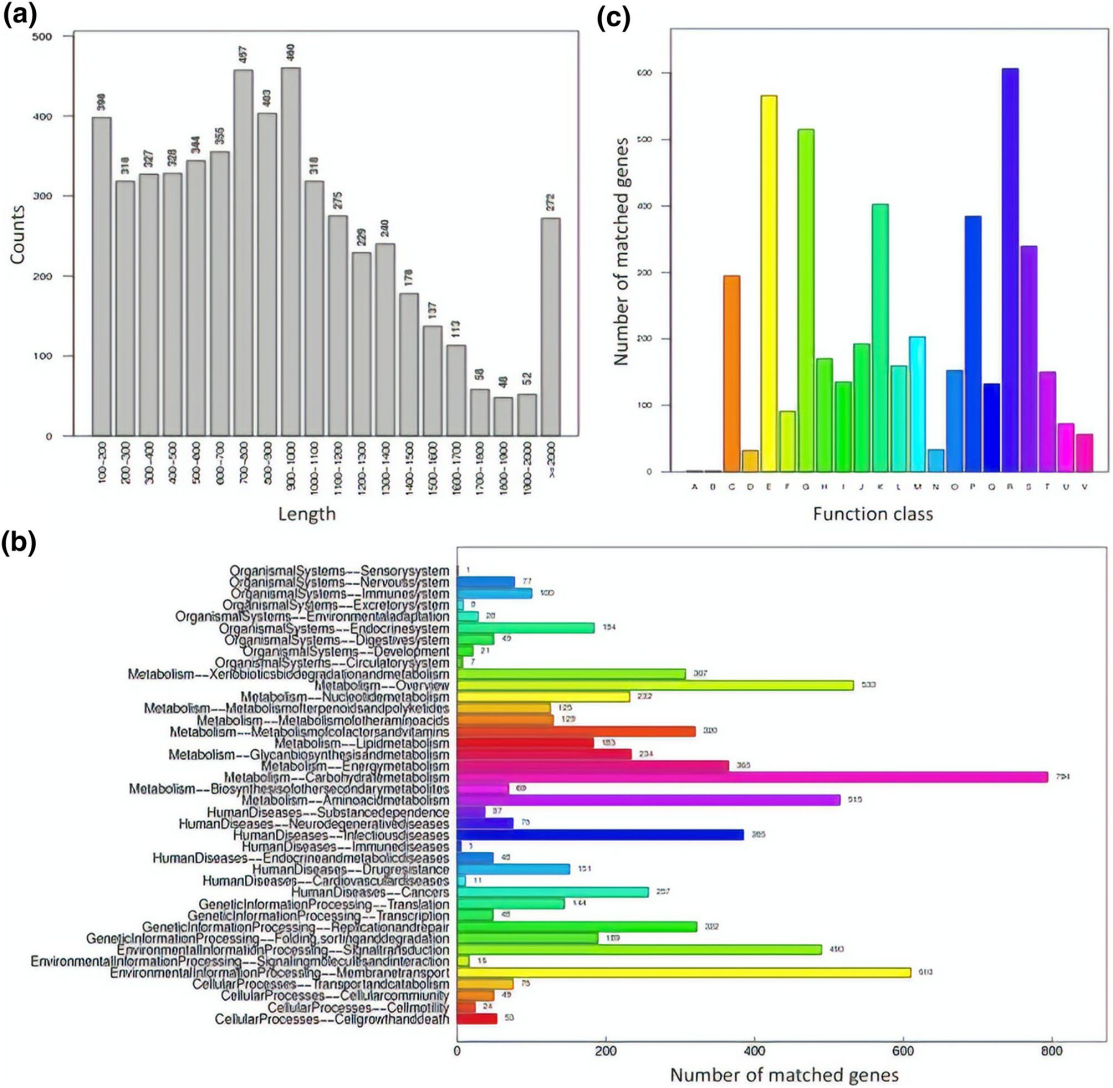
Genome profile of ZK4. **a** Gene length distribution of ZK4; **b** KEGG pathway classification for genome of ZK4; **c** COG classification for genes of ZK4 [A. RNA processing and modification (1); B. Chromatin structure and dynamics (1); C. Energy production and conversion (295); D. Cell cycle control, cell division, chromosome partitioning (32); E. Amino acid transport and metabolism (566); F. Nucleotide transport and metabolism (91); G. Carbohydrate transport and metabolism (515); H. Coenzyme transport and metabolism (170); I. Lipid transport and metabolism (135); J. Translation, ribosomal structure and biogenesis (192); K. Transcription (402); L. Replication, recombination and repair (159); M. Cell wall/membrane/envelope biogenesis (203); N. Cell motility (33); O. Posttranslational modification, protein turnover, chaperones (152); P. Inorganic ion transport and metabolism (384); Q. Secondary metabolism 11 biosynthesis, transport and catabolism (132); R. General function prediction only (606); S. Function unknown (339); T. Signal transduction mechanisms (150); U. Intracellular trafficking, secretion, and vesicular transport (72); V. Defense mechanisms (56)]

After sequencing was completed using the Illumina Hiseq 4000 platform, the genome component analysis showed that the ZK4 sample genome contained 5310 genes with a total length of 4,864,494 bp and an average length of 916 bp. This accounted for 87.19% of the total length of the genome. There were 122 tandem repeats with a total length of 20,718 bp, accounting for 0.3713% of the genome. There are 61 small satellite sequences and ten microsatellite sequences, 85 tRNAs, and 25 rRNAs. The specific results are shown in Table 4 and Fig. 4a.

**Table 4:** Gene prediction results for ZK4

Gene no. (#)	5310
Total length (bp)	4,864,494
Gene average length (bp)	916
Gene length/genome (%)	87.19
GC content in gene region (%)	56.91
Intergenic region length (bp)	714,837
GC content in intergenic region (%)	48.17
Intergenic region length/genome (%)	12.81

### The ZK4 strain KEGG database annotation

Upon comparison with KEGG data, we found that the ZK4 strain gene was divided into 40 categories, each of which had distinct functions and quantities. The specific results are shown in Fig. 4b. The 40 genes, divided into five categories, were, respectively, involved in metabolism, regulation of human diseases, regulation of genetic information, and cellular metabolism. There were 794 genes involved in sugar metabolism, 533 genes involved in exogenous chemical metabolism, and 515 genes involved in amino acid metabolism. The number of transported genes was 610.

### The ZK4 strain genome Clusters of Orthologous Groups database annotation

Clusters of Orthologous Groups (COG) is a database based on the evolutionary relationship of the encoded protein systems of the complete genomes of bacteria, algae, and eukaryotes. By aligning, a particular protein sequence can be annotated into the COG. Each cluster of COGs is composed of orthologous sequences so that the function of the sequence can be inferred.

Compared with the COG database, we found that 4686 protein sequences in the ZK4 strain genome had a COG number, accounting for 88.25% of the total predicted protein. This study clarified features of related chromosomal proteins that corresponded with 22 proteins in the COG database. Additionally, we inferred the function of these chromosomal proteins, and classified them by function; the statistical results are shown in Fig. 4c. Of all the protein functions, the number of general function predictor genes (R) was the highest at 606. Second, more proteins were classified as metabolic, and were involved in amino acid translation and metabolism (E; *n* = 566) and sugar transport and metabolism (G; *n* = 515). These findings indicated that the strain was more metabolically active.

## Discussion

Three strains were used to simultaneously degrade lambda-cyhalothrin and deltamethrin, isolated from soil sediment samples obtained from both sides of the discharge channel of an abandoned pesticide plant in Kaifeng City, Henan Province. The three strains were *Raoultella ornithinolytica* ZK4, *Pseudomonas* sp. ZF1, and *Enterobacter* sp. ZW3. *Pseudomonas* sp. and *Enterobacter* sp. are widely used to degrade pesticides and for microbial remediation. Our results were, therefore, consistent with those of Maloney et al. (1988) and Yu et al. (1996). *Raoultella* sp. has not been applied to the degradation of pyrethroid pesticides and, therefore, has potential research value.

The degrading enzyme produced by the ZK4 strain is located within the cell and is degraded by intracellular enzymes. The degradation rates of the intracellular crude enzyme solution for lambda-cyhalothrin and deltamethrin were 55% and 53%, respectively. The results are basically consistent with the results obtained following intracellular crude enzyme solution treatment of pesticide residues, such as those reported by (Hao et al. 2018; Long et al. 2014; Wang et al. 2005). However, the degradation mechanisms of pesticide-degrading enzymes are poorly understood, and strategies are needed develop related pesticide-degrading enzyme preparations from degrading enzymes. Additionally, we need to understand how to select a flora that can degrade the target pollutants and prepare a mixed microbial agent. Future studies should address these issues.

By sequencing the *Raoultella ornithinolytica* strain, we were able to analyze the number and length of genes contained in the ZK4 genome, the function of annotated genomes, and the number of predicted functional genes. However, we still do not know how to construct a highly resistant engineered strain by cloning a gene fragment that degrades pesticides. We also do not know how engineered strains perform their functions under complex natural conditions. Both of these issues require further in-depth study.

## Conclusion

We separated and screened the ZK4 strain. This strain can simultaneously degrade lambda-cyhalothrin and deltamethrin. We observed the growth of the strain by light microscopy and combined physiological and biochemical tests with 16SrDNA molecular identification. The strain ZK4 was identified as *Raoultella ornithinolytica*. There are no prior examinations of *Raoultella* sp. for degrading pyrethroid pesticides and, therefore, our results have research value.

By performing an enzyme localization study on the ZK4 strain, we found that the degrading enzyme that was produced was an intracellular enzyme. The degradation rates of the intracellular crude enzyme solution for lambda-cyhalothrin and deltamethrin were 55% and 53%, respectively.

We also studied the degradation characteristics of the intracellular crude enzyme solution of the degrading bacteria ZK4 under different conditions. Our results showed that the optimum pH of the two kinds of pyrethroid pesticides was 6.5, and the optimum temperature was 37 °C. At the same time, the intracellular degradation crude enzyme produced by the ZK4 strain exhibited stability to the degradation performance of lambda-cyhalothrin and deltamethrin at a pH of 6.0–8.0 and at a temperature of 20–42 °C.

Sequencing of the ZK4 strain genome revealed 5310 genes with a total length of 4,864,494 bp, and an average length of 916 bp. This accounted for 87.19% of the genome. WE functionally annotated the strain ZK4 genome using the KEGG and COG databases. According to KEGG metabolic pathway analysis, glucose metabolism and exogenous chemical metabolism predominated. When we compared the genome to information obtained from the COG database, it was found that the 4686 protein sequence had a COG number and accounted for 88.25% of the total predicted protein. Among all protein functions, the number of functional predictive genes was the highest, reaching 606. A large number of proteins were classified as metabolic. The numbers of proteins involved in amino acid translation and metabolism and sugar transport and metabolism were relatively large at 566 and 515, respectively.
